# Supplementary material for: Effects of biological sex mismatch on neural progenitor cell transplantation for spinal cord injury in mice
Source: Nat Commun. 2022 Sep 14;13:5380. doi: 10.1038/s41467-022-33134-x (PMC9474813; doi:10.1038/s41467-022-33134-x)
Supplement: Supplementary file 1 — Supplementary Information [file 41467_2022_33134_MOESM1_ESM.pdf]

Supplementary Information

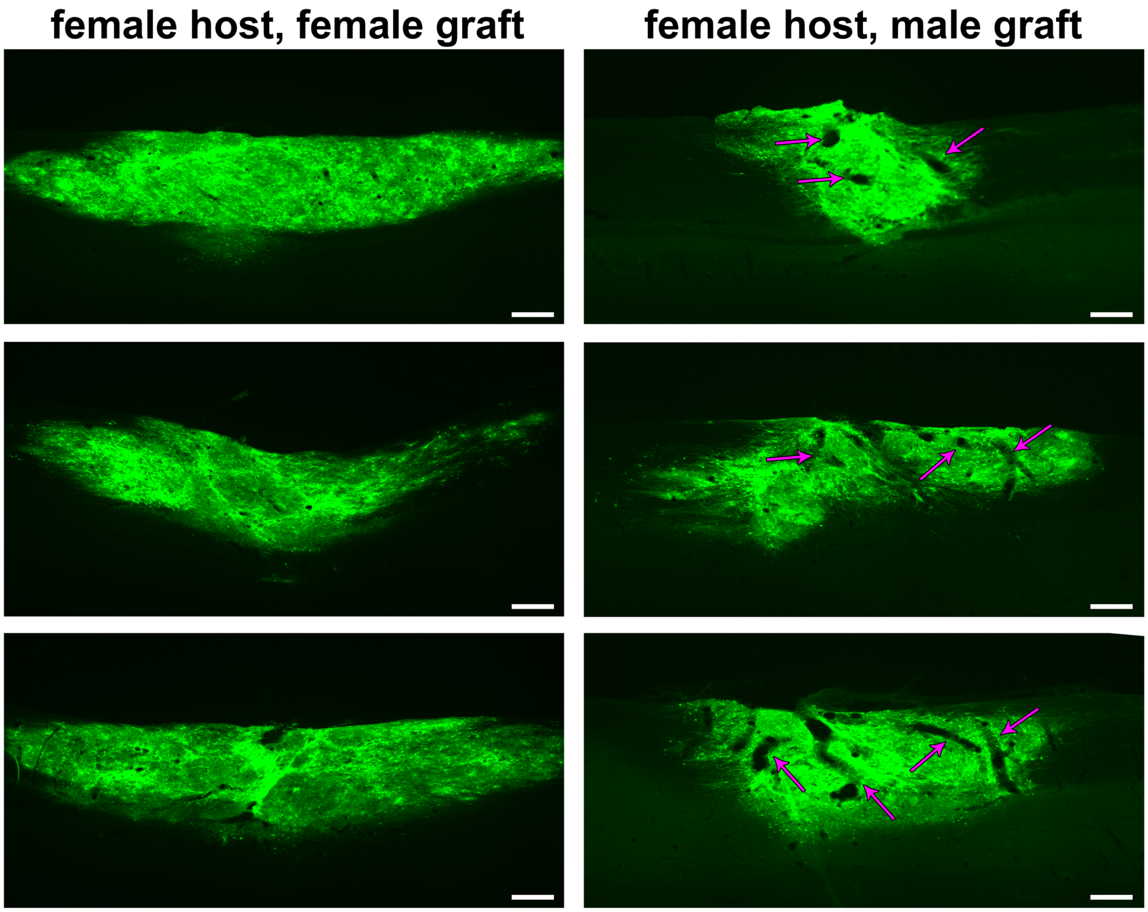

Supplementary Figure 1. Gross appearance of grafts at 4 weeks post-transplantation. The gross appearance of GFP^+ grafts is shown for six individual animals. The left column shows three different subjects from the female host/female graft group, exhibiting typical graft morphology similar to that observed in previous NPC transplantation studies. The right column shows three different subjects from the female host/male graft group; grafts exhibit atypical morphology with large GFP-negative regions resembling vascular structures (arrows). Scale bar = 250 μm. These experiments were performed twice with similar results.

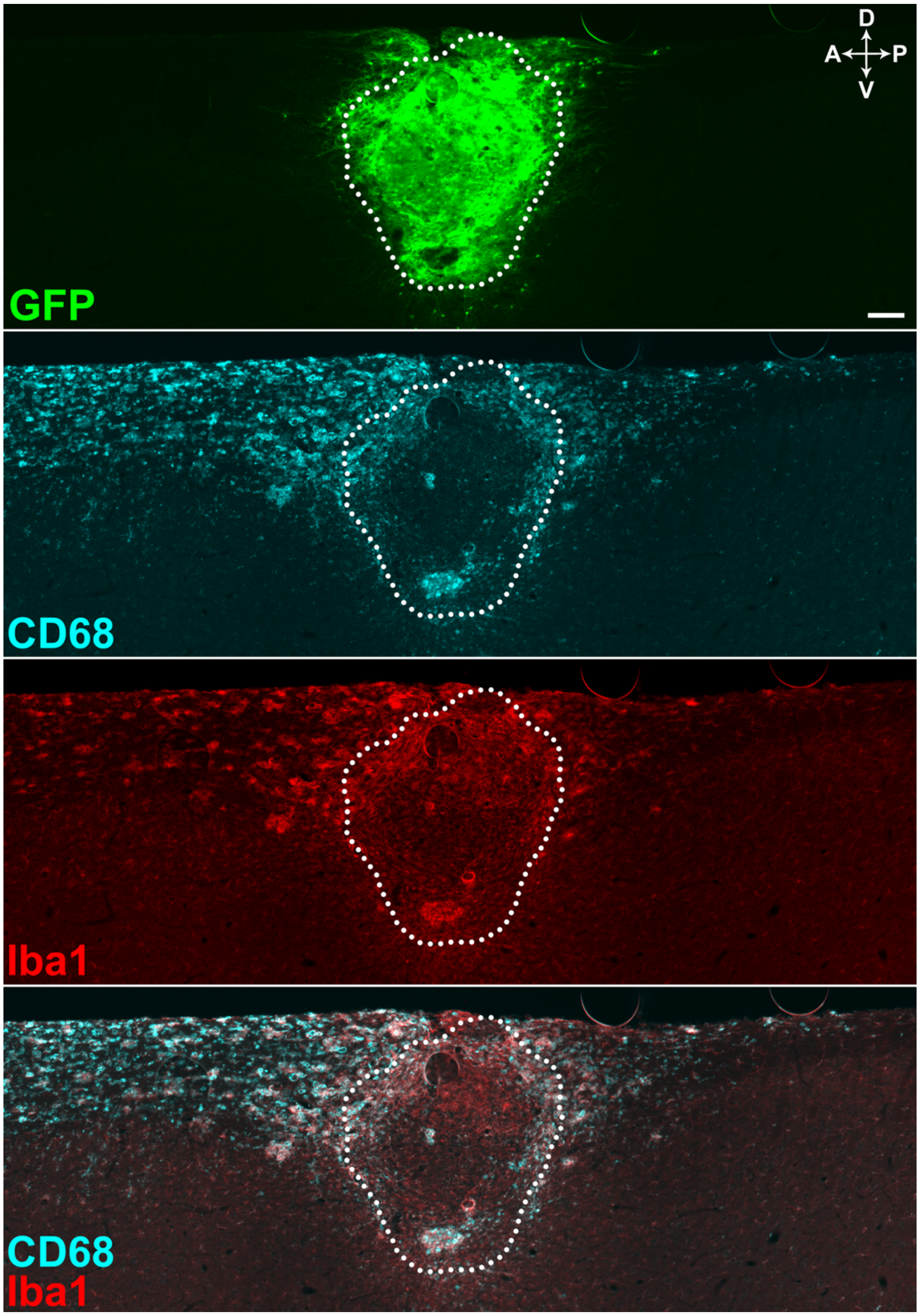

Supplementary Figure 2. Macrophage/microglial immunoreactivity in a neural progenitor cell graft. This image is of a female NPC graft in a female host at 4 weeks post-transplantation. Dotted lines depict the GFP^{+} graft border. CD68 and Iba1 are phagocytic cell markers that label cells within the graft and outside the graft, particularly in the degenerating white matter tracts anterior to the lesion/graft. Scale bar = 100 μm. These experiments were performed twice with similar results.

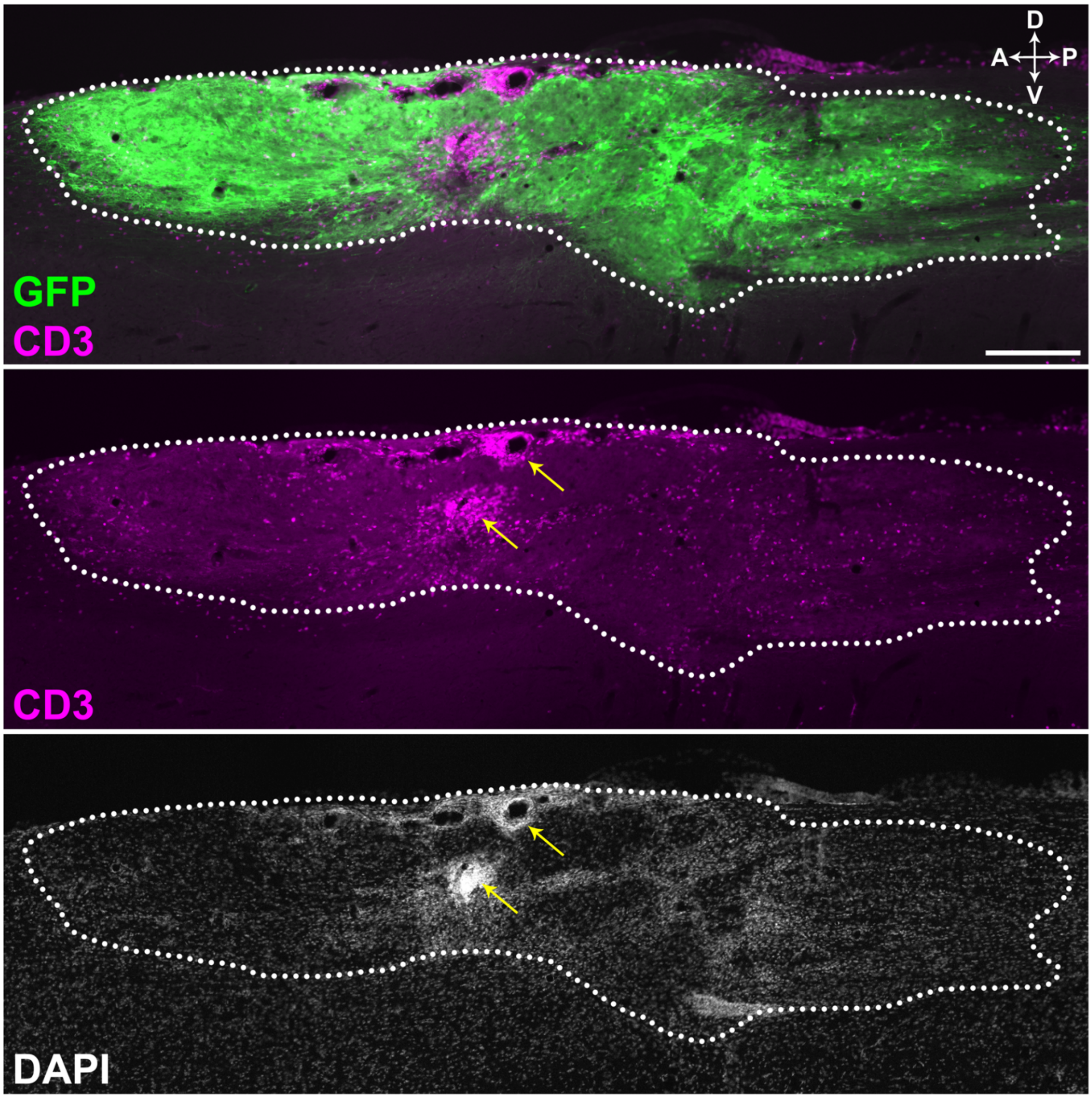

Supplementary Figure 3. T lymphocyte distribution in a neural progenitor cell graft.
This image is of a male NPC graft in a female host at 4 weeks post-transplantation. Dotted lines depict the GFP^{+} graft border. CD3 antigen is a common marker of T lymphocytes; here T cells are seen throughout the graft and concentrated in some perivascular regions (arrows). Scale bar = 250 μm. These experiments were performed twice with similar results.

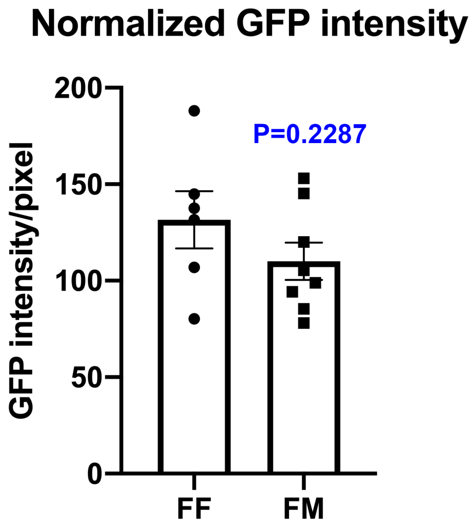

Supplementary Figure 4. Quantification of GFP immunoreactivity in male and female neural progenitor cell grafts. The total intensity of GFP immunoreactivity for male and female grafts within female hosts were quantified. GFP intensity is normalized to graft area. FF (n=6 mice); FM (n=8 mice). All data are mean ± SEM. Significance was determined by an unpaired, two-tailed t test.

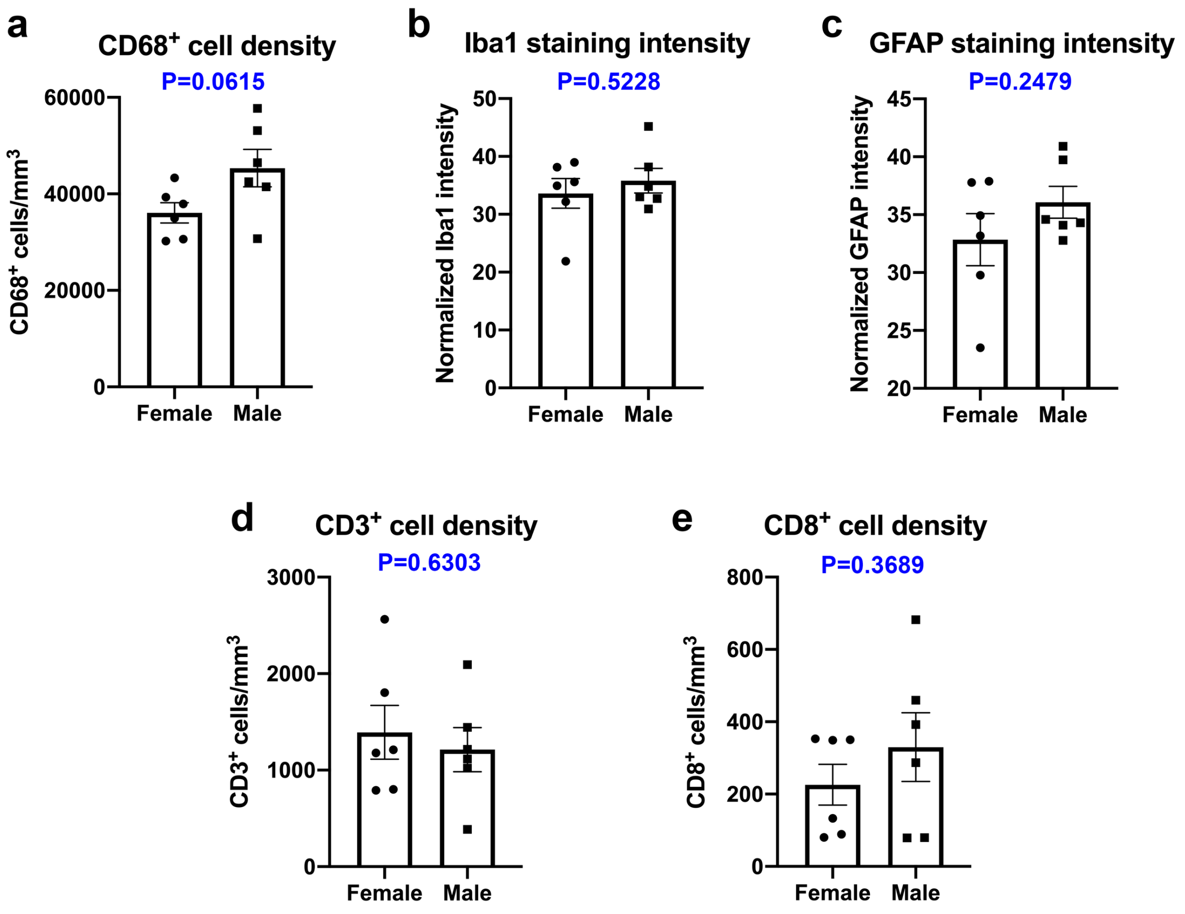

Supplementary Figure 5. Quantification of phagocytic, astroglial, and immune cell markers after spinal cord injury in male and female mice. Data are from male (n=6 mice) and female (n=6 mice) mice at 4 weeks after cervical dorsal column SCI; the lesion site and a 500-μm perilesional area were quantified. Quantifications are of (**a**) the density of $CD68^+$ cells, (**b**) Iba1 immunoreactivity, (**c**) GFAP immunoreactivity, (**d**) $CD3^+$ cell density, and (**e**) $CD8^+$ cell density in and around the lesion site. All data are mean ± SEM. Significance was determined by unpaired, two-tailed t test.

Supplementary Table 1. Detailed description of statistical analyses used in this study.

Figure	Parameter	Group size (N)	Statistical test	Significance level
2b	Graft neuron density	GM (n=5); MM (n=9); MX (n=6); MF (n=9); FF (n=7); FX (n=5); FM (n=10)	Ordinary one-way ANOVA with Dunnett's multiple comparisons test	F (6, 44) = 1.901, P=0.1021
2d	GFAP in graft	MM (n=9); MX (n=8); MF (n=8); FF (n=8); FX (n=6); FM (n=10)	Two-way ANOVA with Sidak's multiple comparisons test	Interaction: F (2, 43) = 0.1007, P=0.9044<br>Graft type: F (2, 43) = 1.226, P=0.3035<br>Host sex: F (1, 43) = 0.7088, P=0.4045<br><br>No significant differences between groups
2e	GFAP around graft	MM (n=9); MX (n=8); MF (n=8); FF (n=8); FX (n=6); FM (n=10)	Two-way ANOVA with Sidak's multiple comparisons test	Interaction: F (2, 43) = 0.4227, P=0.6580<br>Graft type: F (2, 43) = 0.6165, P=0.5446<br>Host sex: F (1, 43) = 1.484, P=0.2298<br><br>No significant differences between groups
2f	Axon outgrowth	MM (n=9); MX (n=5); MF (n=9); FF (n=8); FX (n=6); FM (n=10)	Two-way repeated measures ANOVA with Tukey's multiple comparisons test	Distance x Group main effect: F (95, 779) = 1.981, P<0.0001<br>Distance main effect: F (2.119, 86.89) = 151.7, P<0.0001<br>Group main effect: F (5, 41) = 2.606, P=0.0389<br>Subject main effect: F (41, 779) = 1.93, P<0.0001<br><br>-3500, MM vs. FM: P=0.0271;<br>-3000, MM vs. MX: P=0.0101;<br>-3000, MM vs. FM: P=0.0185;<br>-2500, MM vs. MX: P=0.0329;<br>-2500, MM vs. FM: P=0.0255;<br>-2000, MX vs. MF: P=0.0314;<br>-2000, MX vs. FF: P=0.0363

3c	Vascular footprint	MM (n=9); MX (n=4); MF (n=9); FF (n=5); FX (n=8); FM (n=7)	Two-way ANOVA with Sidak's multiple comparisons test	Interaction: F (2, 36) = 13.45, P<0.0001<br>Graft type: F (2, 36) = 12.52, P<0.0001<br>Host sex: F (1, 36) = 5.472, P=0.0250<br><br>MM vs. FM: P<0.0001;<br>MX vs. FM: P=0.0008;<br>MF vs. FM: P<0.0001;<br>FF vs. FM: P<0.0001;<br>FX vs. FM: P<0.0001
3d	Vascular diameter	MM (n=6); MX (n=4); MF (n=7); FF (n=5); FX (n=8); FM (n=7)	Two-way ANOVA with Sidak's multiple comparisons test	Interaction: F (2, 31) = 4.036, P=0.0277<br>Graft type: F (2, 31) = 9.431, P=0.0006<br>Host sex: F (1, 31) = 1.342, P=0.2554<br><br>MX vs. FM: P=0.0030;<br>FF vs. FM: P=0.0228;<br>FX vs. FM: P=0.0003
3g	Perivascular cell density	MM (n=9); MX (n=4); MF (n=9); FF (n=5); FX (n=6); FM (n=9)	Two-way ANOVA with Sidak's multiple comparisons test	Interaction: F (2, 36) = 4.089, P=0.0251<br>Graft type: F (2, 36) = 7.164, P=0.0024<br>Host sex: F (1, 36) = 3.799, P=0.0591<br><br>MM vs. FM: P=0.0058;<br>MX vs. FM: P=0.0094;<br>MF vs. FM: P=0.0022;<br>FF vs. FM: P=0.0326;<br>FX vs. FM: P=0.0011
4b	Graft $CD68^+$ cell density	MM (n=9); MX (n=7); MF (n=10); FF (n=6); FX (n=6); FM (n=10)	Two-way ANOVA with Sidak's multiple comparisons test	Interaction: F (2, 42) = 0.1519, P=0.8596<br>Graft type: F (2, 42) = 1.601, P=0.2138<br>Host sex: F (1, 42) = 0.1302, P=0.7200<br><br>No significant differences between groups
4c	Graft Iba1 staining intensity	MM (n=8); MX (n=7); MF (n=10); FF (n=6); FX (n=6); FM (n=9)	Two-way ANOVA with Sidak's multiple comparisons test	Interaction: F (2, 40) = 1.054, P=0.3579<br>Graft type: F (2, 40) = 4.136, P=0.0233<br>Host sex: F (1, 40) = 2.354, P=0.1329

				MF vs. FM: P=0.0230
4e	Graft CD3$^+$ cell density	MM (n=9); MX (n=8); MF (n=10); FF (n=7); FX (n=8); FM (n=9)	Two-way ANOVA with Sidak's multiple comparisons test	Interaction: F (2, 45) = 0.5943, P=0.5562<br>Graft type: F (2, 45) = 0.1624, P=0.8506<br>Host sex: F (1, 45) = 0.0005950, P=0.9806<br><br>No significant differences between groups
4f	Graft CD8$^+$ cell density	MM (n=8); MX (n=7); MF (n=9); FF (n=7); FX (n=6); FM (n=8)	Two-way ANOVA with Sidak's multiple comparisons test	Interaction: F (2, 39) = 5.904, P=0.0058<br>Graft type: F (2, 39) = 7.905, P=0.0013<br>Host sex: F (1, 39) = 17.65, P=0.0001<br><br>MM vs. FM: P=0.0001;<br>MX vs. FM: P<0.0001;<br>MF vs. FM: P<0.0001;<br>FF vs. FM: P=0.0002
4g	Perivascular CD3$^+$ cell density	MM (n=9); MX (n=4); MF (n=9); FF (n=5); FX (n=6); FM (n=9)	Two-way ANOVA with Sidak's multiple comparisons test	Interaction: F (2, 36) = 4.542, P=0.0174<br>Graft type: F (2, 36) = 1.011, P=0.3739<br>Host sex: F (1, 36) = 5.727, P=0.0220<br><br>MM vs. FM: P=0.0016;<br>MF vs. FM: P=0.0199
4h	Perivascular CD8$^+$ cell density	MM (n=9); MX (n=4); MF (n=9); FF (n=6); FX (n=6); FM (n=9)	Two-way ANOVA with Sidak's multiple comparisons test	Interaction: F (2, 37) = 4.515, P=0.0176<br>Graft type: F (2, 37) = 7.269, P=0.0022<br>Host sex: F (1, 37) = 6.230, P=0.0171<br><br>MM vs. FM: P=0.0031;<br>MF vs. FM: P=0.0006;<br>FF vs. FM: P=0.0011;<br>MX vs. FM: P=0.0127
4i	% Cytotoxic T cells	MM (n=8); MX (n=7); MF (n=9); FF (n=7); FX (n=6); FM (n=8)	Two-way ANOVA with Sidak's multiple comparisons test	Interaction: F (2, 38) = 3.127, P=0.0553<br>Graft type: F (2, 38) = 6.155, P=0.0048<br>Host sex: F (1, 38) = 13.88, P=0.0006

				MM vs. FM: P=0.0268;<br>MX vs. FM: P=0.0030;<br>MF vs. FM: P=0.0016;<br>FF vs. FM: P=0.0056
S4	Graft GFP intensity	FF (n=6); FM (n=8)	Unpaired t test, two-tailed	t=1.268, df=12<br><br>P=0.2287
S5a	$CD68^+$ cell density	Female (n=6); Male (n=6)	Unpaired t test, two-tailed	t=2.105, df=10<br><br>P=0.0615
S5b	Iba1 staining intensity	Female (n=6); Male (n=6)	Unpaired t test, two-tailed	t=0.6622, df=10<br><br>P=0.5228
S5c	GFAP staining intensity	Female (n=6); Male (n=6)	Unpaired t test, two-tailed	t=1.227, df=10<br><br>P=0.2479
S5d	$CD3^+$ cell density	Female (n=6); Male (n=6)	Unpaired t test, two-tailed	t=0.4964, df=10<br><br>P=0.6303
S5e	$CD8^+$ cell density	Female (n=6); Male (n=6)	Unpaired t test, two-tailed	t=0.9409, df=10<br><br>P=0.3689

Supplementary Table 2. Primary and secondary antibodies used in this study.

Antibody	Catalog #	RRID	Dilution (working concentration)
Primary antibodies			
Rat anti-CD3, clone 17A2	BioLegend #100202	AB_312659	1:100 (5 µg/mL)
Hamster anti-CD31, clone 2H8	Millipore #MAB1398Z	AB_94207	1:500 (1 µg/mL)
Rat anti-CD8a, clone 53-6.7	BioLegend #100702	AB_312741	1:500 (1 µg/mL)
Rat anti-CD68, clone FA-11	BioLegend #137002	AB_2044004	1:100 (5 µg/mL)
Chicken anti-GFAP	Encor Bio #CPCA-GFAP	AB_2109953	1:2000 (5 µg/mL)
Rabbit anti-GFP	Thermo Fisher #A-6455	AB_221570	1:1500 (5 µg/mL)
Rabbit anti-Iba1	Wako #019-19741	AB_839504	1:1000 (5 µg/mL)
Guinea pig anti-NeuN	Millipore #ABN90	AB_11205592	1:3000 (5 µg/mL)
Secondary antibodies			
AlexaFluor 488-AffiniPure Donkey anti-rabbit IgG	Jackson ImmunoResearch #711-545-152	AB_2313584	1:1000 (1 µg/mL)
AlexaFluor 647-AffiniPure Donkey anti-rabbit IgG	Jackson ImmunoResearch #711-605-152	AB_2492288	1:1000 (1 µg/mL)
AlexaFluor 647-AffiniPure Donkey anti-chicken IgY	Jackson ImmunoResearch #703-605-155	AB_2340379	1:1000 (1 µg/mL)
AlexaFluor 488-AffiniPure Donkey anti-guinea pig IgG	Jackson ImmunoResearch #706-545-148	AB_2340472	1:1000 (1 µg/mL)
AlexaFluor 594-AffiniPure Donkey anti-guinea pig IgG	Jackson ImmunoResearch #706-585-148	AB_2340474	1:1000 (1 µg/mL)
AlexaFluor 594-AffiniPure Goat anti-rat IgG	Jackson ImmunoResearch #112-585-062	AB_2338377	1:1000 (1 µg/mL)
AlexaFluor 647-AffiniPure Goat anti-rat IgG	Jackson ImmunoResearch #112-605-062	AB_2338398	1:1000 (1 µg/mL)
AlexaFluor 647-AffiniPure Goat anti-Armenian hamster IgG	Jackson ImmunoResearch #127-605-160	AB_2339001	1:1000 (1 µg/mL)
