# Supplementary material for: Effects of biological sex mismatch on neural progenitor cell transplantation for spinal cord injury in mice
Source: Nat Commun. 2022 Sep 14;13:5380. doi: 10.1038/s41467-022-33134-x (PMC9474813; doi:10.1038/s41467-022-33134-x)
Supplement: Supplementary file 3 — Reporting summary [file 41467_2022_33134_MOESM3_ESM.pdf]

nature portfolio

Corresponding author(s): Jennifer Dulin

Last updated by author(s): Aug 26, 2022

Reporting Summary

Nature Portfolio wishes to improve the reproducibility of the work that we publish. This form provides structure for consistency and transparency in reporting. For further information on Nature Portfolio policies, see our Editorial Policies and the Editorial Policy Checklist.

Statistics

For all statistical analyses, confirm that the following items are present in the figure legend, table legend, main text, or Methods section.

n/a	Confirmed	
☐	☒	The exact sample size (*n*) for each experimental group/condition, given as a discrete number and unit of measurement
☐	☒	A statement on whether measurements were taken from distinct samples or whether the same sample was measured repeatedly
☐	☒	The statistical test(s) used AND whether they are one- or two-sided<br>*Only common tests should be described solely by name; describe more complex techniques in the Methods section.*
☐	☒	A description of all covariates tested
☐	☒	A description of any assumptions or corrections, such as tests of normality and adjustment for multiple comparisons
☐	☒	A full description of the statistical parameters including central tendency (e.g. means) or other basic estimates (e.g. regression coefficient) AND variation (e.g. standard deviation) or associated estimates of uncertainty (e.g. confidence intervals)
☐	☒	For null hypothesis testing, the test statistic (e.g. *F*, *t*, *r*) with confidence intervals, effect sizes, degrees of freedom and *P* value noted<br>*Give P values as exact values whenever suitable.*
☒	☐	For Bayesian analysis, information on the choice of priors and Markov chain Monte Carlo settings
☒	☐	For hierarchical and complex designs, identification of the appropriate level for tests and full reporting of outcomes
☒	☐	Estimates of effect sizes (e.g. Cohen's *d*, Pearson's *r*), indicating how they were calculated

Our web collection on statistics for biologists contains articles on many of the points above.

Software and code

Policy information about availability of computer code

Data collection	Nikon NIS Elements software (AR 5.02.00) was used for image acquisition.
Data analysis	ImageJ 1.53h was used for data analysis.

For manuscripts utilizing custom algorithms or software that are central to the research but not yet described in published literature, software must be made available to editors and reviewers. We strongly encourage code deposition in a community repository (e.g. GitHub). See the Nature Portfolio guidelines for submitting code & software for further information.

Data

Policy information about availability of data

All manuscripts must include a data availability statement. This statement should provide the following information, where applicable:

- Accession codes, unique identifiers, or web links for publicly available datasets
- A description of any restrictions on data availability
- For clinical datasets or third party data, please ensure that the statement adheres to our policy

Data sharing not applicable to this article as no datasets were generated or analysed during the current study.

Human research participants

Policy information about studies involving human research participants and Sex and Gender in Research.

Reporting on sex and gender	*Use the terms sex (biological attribute) and gender (shaped by social and cultural circumstances) carefully in order to avoid confusing both terms. Indicate if findings apply to only one sex or gender; describe whether sex and gender were considered in study design whether sex and/or gender was determined based on self-reporting or assigned and methods used. Provide in the source data disaggregated sex and gender data where this information has been collected, and consent has been obtained for sharing of individual-level data; provide overall numbers in this Reporting Summary. Please state if this information has not been collected. Report sex- and gender-based analyses where performed, justify reasons for lack of sex- and gender-based analysis.*
Population characteristics	*Describe the covariate-relevant population characteristics of the human research participants (e.g. age, genotypic information, past and current diagnosis and treatment categories). If you filled out the behavioural & social sciences study design questions and have nothing to add here, write "See above."*
Recruitment	*Describe how participants were recruited. Outline any potential self-selection bias or other biases that may be present and how these are likely to impact results.*
Ethics oversight	*Identify the organization(s) that approved the study protocol.*

Note that full information on the approval of the study protocol must also be provided in the manuscript.

Field-specific reporting

Please select the one below that is the best fit for your research. If you are not sure, read the appropriate sections before making your selection.

☒ Life sciences ☐ Behavioural & social sciences ☐ Ecological, evolutionary & environmental sciences

For a reference copy of the document with all sections, see nature.com/documents/nr-reporting-summary-flat.pdf

Life sciences study design

All studies must disclose on these points even when the disclosure is negative.

Sample size	A power analysis was performed to determine sample size, using a desired power of 0.8 and alpha level of 0.05.
Data exclusions	Data were excluded from analysis of immunohistochemical images if samples exhibited poor immunostaining quality, or if samples did not contain sections with clear graft tissue visible.
Replication	To verify reproducibility, two different experimental cohorts were generated. Immunohistochemical experiments were performed twice. Genotyping was performed three times. All attempts at replication were successful.
Randomization	Animal subjects were randomly assigned to receive male, female, or mixed NPC grafts using a random number generator internet site.
Blinding	All investigators were blinded to the treatment during animal experiments and data analysis.

Reporting for specific materials, systems and methods

We require information from authors about some types of materials, experimental systems and methods used in many studies. Here, indicate whether each material, system or method listed is relevant to your study. If you are not sure if a list item applies to your research, read the appropriate section before selecting a response.

Materials & experimental systems

n/a	Involved in the study	
☐	☒	Antibodies
☒	☐	Eukaryotic cell lines
☒	☐	Palaeontology and archaeology
☐	☒	Animals and other organisms
☒	☐	Clinical data
☒	☐	Dual use research of concern

Methods

n/a	Involved in the study	
☒	☐	ChIP-seq
☒	☐	Flow cytometry
☒	☐	MRI-based neuroimaging

Antibodies

Antibodies used	Primary antibodies: Rat anti-CD3 (clone 17A2, BioLegend #100202), Hamster anti-CD31 (clone 2H8, Millipore #MAB1398Z), Rat anti-CD8a (clone 53-6.7, BioLegend #100702), Rat anti-CD68 (clone FA-11, BioLegend #137002), Chicken anti-GFAP (EncorBio #CPCA-GFAP), Rabbit anti-GFP (Thermo Fisher #A-6455), Rabbit anti-Iba1 (Wako #019-19741), Guinea pig anti-NeuN (Millipore #ABN90). Secondary antibodies: AlexaFluor-conjugated secondary antibodies from Jackson Immunoresearch were used for all experiments. This includes the following catalog numbers: #711-545-152, 711-605-152, 703-605-155, 706-545-148, 706-585-148, 112-585-062, 112-605-062, 127-605-160.
Validation	Each primary antibody was validated in mouse spinal cord tissue sections by using a secondary-only control condition (in which no primary antibody was applied, but the appropriate species of fluorescently-conjugated secondary antibody was used), and confirming lack of fluorescent signal. Antibodies were also validated by ensuring that patterns of immunoreactivity in mouse spinal cord tissue are similar to previously published historical controls.

Animals and other research organisms

Policy information about studies involving animals; ARRIVE guidelines recommended for reporting animal research, and Sex and Gender in Research

Laboratory animals	C57BL/6 mice (Jackson laboratories #000664), male and female, 8-10 weeks old; GFP mice (Jackson laboratories #006567), female, 8-10 weeks old. Animals had free access to food and water throughout the study and were group-housed in standard Plexiglas cages on a 12-hour light / 12-hour dark cycle (light cycle = 6:00 am – 6:00 pm). Animal housing facilities had ambient temperature between 20-23°C and 30-70% humidity.
Wild animals	The study did not utilize wild-caught animals.
Reporting on sex	Sex of animals was a central feature of study design; as such, sex-based analysis are provided throughout the entire manuscript.
Field-collected samples	The study did not utilize samples collected from the field.
Ethics oversight	All animal experiments were approved by the Texas A&M University Institutional Animal Care and Use Committee.

Note that full information on the approval of the study protocol must also be provided in the manuscript.
